# Rapid and specific detection of *Enterococcus faecalis* with a visualized isothermal amplification method

**DOI:** 10.3389/fcimb.2022.991849

**Published:** 2022-09-12

**Authors:** Bo Zhu, Juan Hu, Xuelian Li, Xiaomin Li, Lei Wang, Shihui Fan, Xin Jin, Kun Wang, Weiguo Zhao, Wenjun Zhu, Cheng Chen, Zilu Wang, Yingzhi Lu

**Affiliations:** ^1^ Department of Laboratory Medicine, Affiliated Hospital of Jiangsu University, Zhenjiang, China; ^2^ Department of Medicine Laboratory, The Second People's Hospital of Lianyungang (Cancer Hospital of Lianyungang), Lianyungang, China; ^3^ Department of Medicine Laboratory, The Fourth People's Hospital of Lianyungang, Lianyungang, China; ^4^ Department of Oncology, Lianyungang Second People’s Hospital Affiliated to Bengbu Medical College (Lianyungang Hospital Affiliated to Jiangsu University), Lianyungang, China; ^5^ School of Biotechnology, Jiangsu University of Science and Technology, Zhenjiang, China; ^6^ Vascular Surgery, The Second People's Hospital of Lianyungang (Cancer Hospital of Lianyungang), Lianyungang, China

**Keywords:** *Enterococcus faecalis*, recombinase polymerase amplification, lateral flow strip, molecular detection, qPCR

## Abstract

*Enterococcus faecalis* is a serious problem for hospitals and can spread from patient to patient. Most of the current detection methods are associated with limitations associated with the need for trained personnel; they are also time-consuming. Thus, it is necessary to develop rapid and accurate detection methods to control the spread of *E. faecalis.* In this study, we developed a rapid and accurate detection method for *E. faecalis* using recombinase polymerase amplification (RPA) combined with a lateral flow strip (LFS). This method could be completed in approximately 35 min at 37°C. The limit of detection was 10 CFU/µL, irrespective of whether the templates were pure or complex. This method also showed good specificity and compatibility. In total, 278 clinical samples were tested using the RPA-LFS method; the detection accuracy was equal to that of the conventional qPCR method. This visualized isothermal amplification method could be useful for the future on-site detection of *E. faecalis*.

## Introduction

*Enterococcus faecalis* (*E. faecalis*) is a form of Gram-positive bacterium that can withstand oxidative stress, desiccation, extremes of temperature, high ionic strength and alkaline pH (Klare et al., 2001). Furthermore, *E. faecalis* can grow in temperatures between 10−45°C, survive up for 30 min at 60°C and also resist antimicrobial agents; these characteristics differ from the majority of Gram-positive bacteria (Li and Nikaido, 2009; Endo et al., 2015). *E. faecalis* has been determined to be an important opportunistic pathogen and may cause urinary tract infections, bacteremia, infective endocarditis and various other infections (Tailor et al., 1993; Oesterle et al., 2019). Importantly, *E. faecalis* can cause different types of infections, involving clinical instruments, health care workers and may spread from patient to patient (Siegel et al., 2007). *E. faecalis* also accounts for 80% of hospital-acquired *Enterococcus* infections in the USA (Gordana et al., 2018). Consequently, *E. faecalis* is now considered as an important risk factor for patients and hospitals.

For patients and hospitals, accurate pathogen diagnosis is very important for disease prevention and selecting the appropriate treatment option (Agashe et al., 2009). Thus far, many useful methods has been established for the detection of clinical pathogens, including polymerase chain reaction (PCR), quantitative PCR (qPCR), biochemical analysis, conventional culture procedures and immunological-based diagnostic tests (Haugland et al., 2005; He and Jiang, 2005; Kim et al., 2011; Yen et al., 2014). Although many different methods have been developed to detect *E. faecalis*, many of these methods also have shortcomings that we cannot ignore. Most of these tests can be performed well in well-equipped laboratories, but outside of the laboratory, these tests are difficult if not impossible to perform accurately and efficiently in the residences of patients or poorly equipped hospitals (Fukuta et al., 2003). In addition, trained personnel and complicated sample preparation are also required; these steps are also time-consuming. Due to the high prevalence and the difficulties involved in diagnosing *E. faecalis* infection in an accurate manner, it is clear that we need to develop an on-site diagnosis test with a rapid response time, a simple procedure, and with low demand for resources (Xia et al., 2014; Nazari-Vanani et al., 2019). Over recent years, many isothermal amplification methods have been rapidly developed, including loop-mediated isothermal amplification (LAMP) and recombinase polymerase amplification (RPA) (Wu et al., 2018; Wang et al., 2022a).

RPA technology can be completed in a short period of time and therefore represents a suitable choice for the on-site detection of diseases (Zhao et al., 2021; Li et al., 2021). RPA technology relies on recombinase (UvsX and UvsY), single-stranded binding protein (gp32), and strand displacing DNA polymerase (Bsu) for nucleic acid amplification at a low temperature, usually 37°C, thus eliminating the dependency on a sophisticated thermocycler (Piepenburg et al., 2006). RPA amplicons can be detected by gel electrophoresis, a fluorescence detector and by lateral flow strips (LFSs) (Khater et al., 2019; Ma et al., 2021; Wang et al., 2022b). By applying these methods, the results of RPA-LFS can be analyzed visually without the need for trained personnel. This visualization depends on a fluorescein isothiocyanate (FITC) label at the 5’ end, a C3 spacer (SpC3) label at the 3’ end of the probe, and a base is also replaced by tetrahydrofuran (THF) in the middle of the probe. If the probe binding to an amplified strand, the Nfo cutting at the THF site can be initiated to release the 3’ end of the probe for an extension.Because the reverse primer was labeled with a biotin, the amplification product has FITC and biotin labels at the two ends. The gold nanoparticle-tagged (AuNP-tagged) anti-FITC antibody on the conjugate pad of LFSs can bind to the amplification product. This product can be captured by the streptavidin-coated test line and aggregated, and the red color positive signal is exhibited.

In this study, a simple and rapid detection method for *E. faecalis* was established by designing specific primers and probes. This is a unique tool for detecting *E. faecalis* in sputum. This method can confirm *E. faecalis* infection and provide patients with early intervention and appropriate therapeutic options.

## Materials and methods

### Bacteria strains and DNA extraction

In this study, *E. faecalis, Acinetobacter baumannii, Candida parapsilosis, Candida tropicalis* and *Candida albicans* were purchased from the American Type Culture Collection (Manassas, VA, USA). In addition, isolates of *E. faecalis* strains, isolates of other *Enterococcus* species, and isolates of other infectious pathogens were provided by The Second People’s Hospital of Lianyungang (Lianyungang, China). Sputum-isolated strains and clinical samples of sputum were collected from patients; 16S rRNA sequencing was performed to confirm the strains as described previously (Hiergeist et al., 2015). Information relating to the strains is given in **Table 1**. All strains were incubated in Luria–Bertani broth at 37°C with shaking at 200 rpm for 6 hrs. Bacterial cells were treated at 100°C for 10 min. Furthermore, 1 µL of each bacterial culture was used as a template for the detection of RPA. If purified DNA was used, DNA was extracted from the bacteria using a TIANamp Genomic DNA Kit (Tiangen Biotech Co. Ltd, Beijing, China) and quantified by a Qubit 4 system (Thermo Fisher Scientific Inc, Wilmington, DE, USA).

**Table 1:** The bacterial strains used in this study.

Species	Source	Strain designation
*E. faecalis*	Reference strain	ATCC 19433
*E. faecalis*	Reference strain	ATCC 29212
*E. faecalis*	Reference strain	ATCC 33186
*E. faecalis*	Reference strain	ATCC 49452
*E. faecalis*	Reference strain	ATCC 51299
*E. faecalis*	Reference strain	ATCC 7080
*E. faecalis*	Sputum isolated strain	#1 #2 #3 #4 #5 #6 #7 #8 #9 #10 #11 #12 #13 #14 #15 #16 #17 #18 #19
*E. cloacae*	Sputum isolated strain	N/A
*E. faecium*	Sputum isolated strain	N/A
*E. coli* O157	Sputum isolated strain	N/A
*A. baumannii*	Reference strain	ATCC 19606
*A. fumigatus*	Sputum isolated strain	N/A
*A. calcoaceticus*	Sputum isolated strain	N/A
*A. lwoffi*	Sputum isolated strain	N/A
*A. haemolytius*	Sputum isolated strain	N/A
*A. junii*	Sputum isolated strain	N/A
*C. parapsilosis*	Reference strain	ATCC 22019
*C. tropicalis*	Reference strain	ATCC 20962
*C. albicans*	Reference strain	ATCC 10231
*C. auris*	Sputum isolated strain	N/A
*C. dubliniensis*	Sputum isolated strain	N/A
*C. glabrata*	Sputum isolated strain	N/A
*C. neoformans*	Reference strain	ATCC 14116
*S. aureus*	Sputum isolated strain	N/A
*S. capitis*	Sputum isolated strain	N/A
*S. epidermidis*	Sputum isolated strain	N/A
*S. haemolyticus*	Sputum isolated strain	N/A
*S. hominis*	Sputum isolated strain	N/A
*S. saprophyticus*	Sputum isolated strain	N/A
*S. warneri*	Sputum isolated strain	N/A
*S. maltophilia*	Sputum isolated strain	N/A
*S. pneumonia*	Sputum isolated strain	N/A
*V. streptococci*	Sputum isolated strain	N/A

ATCC, American Type Culture Collection (Manassas, VA, USA). NA, Not applicable.

Clinical samples of sputum were treated at 100°C for 10 min. Furthermore, 1 µL of each samples was used as a template for the detection of RPA-LFS and qPCR methods.

### Design of primers and probes

In order to ensure the specificity of the detection, we screened the genome of *E. faecalis* and identified a DNA fragment (GenBank: AAO79909.1) as the target gene for the RPA assay. The FASTA sequence of the fragment was uploaded into the NCBI Primer-BLAST website (https://www.ncbi.nlm.nih.gov/tools/primer-blast/) to screen the appropriate forward and reverse primers. The key parameter settings were as follows: product size: 100 to 250; primer size: 30 to 34; GC content: 20 to 80; other parameters were applied at default settings.

In order to reduce the influence of the probe on subsequent amplification, we used the forward primer that was used in the previous screening for use as an important part of the probe. Then, we screened the new forward primers in the 5´ section of the probe. The probe was designed using Primer Premier 5 software. The key parameters were as follows: size of probe: 45 to 48 nucleotides; melting temperature (*Tm*): 50 to 100°C; GC content: 20 to 70. In addition, if the probes and primers had three consecutive matching bases, then the probes were mutated to avoid false-positive results. All of the primers and probe used in this research are given in **Table 2**.

**Table 2:** Primers and probes.

Primers/Probes	Primer Sequences	Size (bp)	Reaction name
EF-F1	GTGGCAGTTTTCCTATTTGTACTGATTTTG	30	RPA
EF-R1	GCTAAATCCTGTCCATCCAGATTCTTAACT	30	
EF-F2	AGGCGTTGTTTGTCTCAACAGCCACTATTTC	31	
EF-R2	CGCTCTGCACCGATTGGACGATTGCGTATTT	31	
EF-P	FITC-GTGGCAGCTTTCCTATTTGTACTGCTTTTG[THF]ACGAAAGCTGACATT-C3 spacer	45	RPA-LFS
EF-R1B	Biotin-GCTAGATCCTATCCATCCAGATTCTTAACT	30	
EF-F3	GTCTCAACAGCCACTATTTCTCGGACAGCA	30	
EF-F4	AATGATTGAGCGTGCGAATACGATTGAAGT	30	
EF-F5	ATGACGGAAATTAGTTCACAAACCATTTATGAGG	34	
EF-F6	CGGAAATTAGTTCACAAACCATTTATGAGG	30	
EF-F7	TTGTTTGTCTCAACAGCCACTATTTCTCGG	30	
qPCR-F	TGTTCGGTGTTGGTG	15	qPCR
qPCR-R	ACTGCTGCCGCTTGT	15	

F, forward primer; R, reverse primer; P, probe.

### RPA-LFS procedure

Using templates and the primers described in Section 2.2, we prepared a reaction system in accordance with the manufacturer’s instructions of the Twist Amp^®^ DNA Amplification Kit (TwistDx Ltd., Maidenhead, UK). Each 50 μL reaction mixture contained 2.1 μL of each primer (10 μM), 0.6 μL of probe and 1 μL of template; other standard reaction components were also added to the lid and the reaction was performed at 37°C for 30 min. Then, 10 μL of the RPA amplification product was spotted on a LFS (Ustar Biotechnologies Ltd., Hangzhou, China). The LFS was composed of a sample pad, conjugate pad (soaked with a monoclonal AuNP-tagged anti-FITC antibody), a test line (coated with streptavidin), a control line (coated with anti-mouse antibody) and an absorbent pad that lined up through the solvent migration route. The RPA amplification product was added to the sample pad of the LFS and the stick of the LFS was inserted into 100 µL of the solvent for approximately 5 min.

### The qPCR procedure

The qPCR for *E. faecalis* was performed as previously reported (Sedgley et al., 2004). The forward primer was named qPCR-F (TGTTCGGTGTTGGTG) and the reverse primer was named qPCR-R (ACTGCTGCCGCTTGT). The DNA templates used in qPCR were extracted with a Magnetic Universal Genomic DNA Kit (Tiangen Biotech Co., Ltd.).

## Results

### Design and screening of RPA primers and probes for the detection of *E. faecalis*

**Figure 1:**
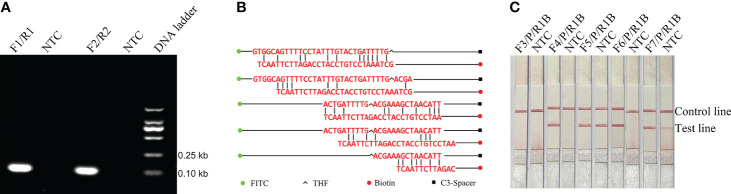
Amplification performance of the primers and probe. **(A)** Image of an agarose gel showing the RPA results of primer pairs targeting a specific fragment. The name of primer pairs are indicated at the top of each lane. NTC, no template control. The image represents the results of three independent experiments. **(B)** Pairing analysis and sequence modifications of the reverse primer-probe sets for the detection of *E. faecalis* using Primer Premier 5 software. Relevant DNA bases of the probe and reverse primer had been excluded. All the DNA strands are shown as horizontal lines and matching bases are indicated by vertical lines. Molecular markers are listed under the figure. **(C)** Screening of the forward primers. Image showing the RPA-LFS results for different forward primers. The names of the primer-probe pairs are indicated at the top of each lane. NTC, No Template Control. The image represents the results of three independent experiments.

Analysis showed that RPA amplification with the two sets of primer pairs did not result in the production of nonspecific bands and the amplicons were of the expected size. These results showed that the two sets of primer pairs have good specificity for the detection of *E. faecalis* (**Figure 1A**). In order to introduce the probe into the RPA-LFS system without causing unnecessary amplification, the forward primer (EF-F1) was changed to the probe and potential mismatch was analyzed using specific software (**Figure 1B**). The RPA reaction has previously been shown to tolerate a few mismatches between the primer/probe and the template (Daher et al., 2016; Wu et al., 2018). In order to reduce the possibility of non-specific amplification, the matched bases for the reverse primer and probe were changed (marked in red in **Table 2**). In addition, five new forward primers were designed at the 5´ end of the probe; these were combined with the modified probe-primer pair for RPA-LFS detection. Results showed that F6-P-R1B exhibited good amplification ability in the RPA-LFS (**Figure 1C**).

### Detection specificity of the RPA-LFS method

**Figure 2:**
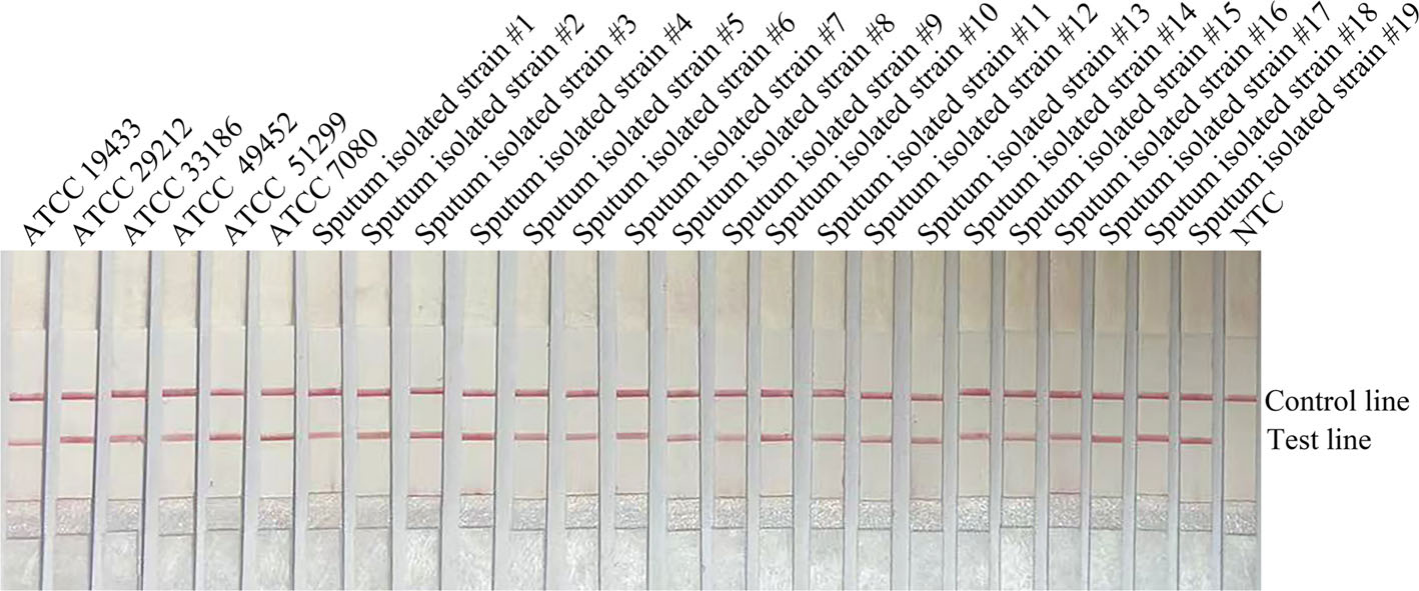
Compatibility confirmation. Image showing the RPA-LFS results for different reference and isolated strains of *E. faecalis*. The name of each bacterial template is indicated on the top of each lane. NTC, no template control. Image represents the results of three independent experiments.

**Figure 3:**
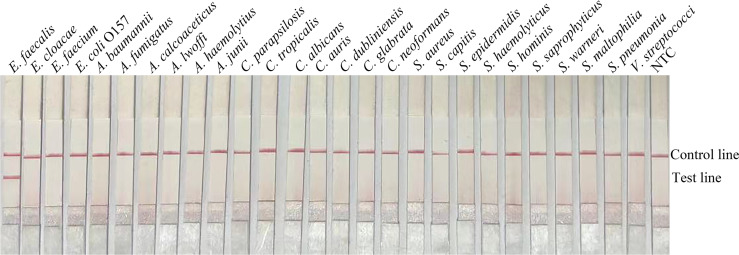
Specificity confirmation. Image showing the RPA-LFS results of different bacterial templates. The species name of the bacterial templates is shown on top of each lane. NTC, no template control. Image represents the results of three independent experiments.

In order to confirm that the detection method could effectively detect different sources of *E. faecalis*, we detected six types of reference strain and 19 types of sputum-isolated strains with the primer-probe set F6-P-R1B; all samples were proven to be positive (**Figure 2**). To evaluate the specificity of the RPA-LFS method, 26 types of common pathogens were detected; only *E. faecalis* was positive. All other strains were negative, thus indicating good specificity (**Figure 3**). The remainder of our research completed with the primer-probe set F6-P-R1B.

### Limit of detection for the RPA-LFS method

**Figure 4:**
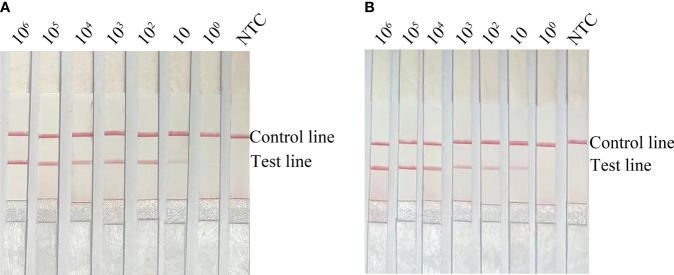
Limit of detection for the RPA-LFS method. **(A)** Image showing detection results with different amounts (10^6^ to 10^0^ CFU/µL) of *E*. *faecalis*. **(B)** Image showing detection results for different amounts (10^6^ to 10^0^ CFU/µL) of *E*. *faecalis*, 10^6^ CFU/µL of *E*. *coli* O157 and 10^6^ CFU/µL of *E*. *faecium.* All reactions were completed at 37°C. NTC: no template control. Image represents the results of three independent experiments.

If using pure *E. faecalis* as a template, the limit of detection (LOD) of the RPA-LFS method was 10 CFU/µL (**Figure 4A**). To simulate a complex sample environment, pure *E. faecalis* was spiked with 10^6^ CFU/µL of *E. coli* O157 and 10^6^ CFU/µL of *E. faecium.* Collectively, these results showed that the RPA-LFS method could tolerate the influence of other bacteria and that the LOD was 10 CFU/µL (**Figure 4B**).

### Application of the RPA-LFS method for the detection of *E. faecalis*

Clinical samples were obtained from The Second People’s Hospital of Lianyungang (Lianyungang, China). The clinical application of the RPA-LFS method for detecting *E. faecalis* was evaluated with 278 clinical samples. The detection results of RPA-LFS were then compared with conventional qPCR and showed 100% consistency (**Table 3**), and the data of RPA-LFS and qPCR were lised in **Supplementary Table S1**. These results demonstrated that the RPA-LFS method represents a good alternative to qPCR for the on-site diagnosis of EF.

**Table 3:** The prevalence of *E. faecalis* in 278 clinical isolates with the RPA-LFS and qPCR assays (summarized).

		RPA-LFS assay		
		Positive	Negative	Total
qPCR	Positive	93	0	93
	Negative	0	185	185
	Total	93	185	278

## Discussion

*E. faecalis* is a serious threat to the global community and can cause urinary tract infections, bacteremia, infective endocarditis and various other infections (Tailor et al., 1993; Oesterle et al., 2019). Currently, many detection methods require long periods of time to obtain results, such as the conventional culture method. Thus, a rapid and sensitive detection method can identify *E. faecalis* infection during the early stages period; this is a very important clinical feature. RPA detection methodology can make up for the shortcomings of the existing detection methods and when compared with other isothermal amplification technologies. In samples of water taken from the environment, such as agricultural wells on animal farms, coastal waters, rivers, and canals, the fecal contaminants are mainly *E. faecalis* (Malani et al., 2002). Furthermore, the components in samples from such sources tend to be very complex. Fortunately, RPA is well tolerated for crude templates and does not require strict temperature control equipment; RPA reactions can even be completed using human body heat (Piepenburg et al., 2006); consequently, RPA technology is useful for the farm and in the clinic.

The RPA-LFS method showed excellent detection efficiency for *E. faecalis*. The LOD was 10 CFU/µL and was comparable to that of the quantitative PCR method which has sensitivities in the range of 10^1^ - 10^2^ CFU (Sedgley et al., 2005). However, the RPA-LFS method could complete reactions in 35 min; the PCR or qPCR method may require over 50 min. Furthermore, when equipment was lacking, there may be a significant time delay caused by the transportation of samples to appropriate laboratories with the equipment necessary for qPCR.

In this study, the primer-probe set for the target gene was evaluated by NCBI Nucleotide BLAST; all 100 of the returned hits were *E. faecalis.* None of the returned sequences were from other species. These results indicated that the primer-probe set F6-P-R1B was highly specific to *E. faecalis*. Of the *Enterococcal* species, only *E. faecalis* and *Enterococcus faecium* commonly colonize and infect human in detectable numbers (Kim et al., 2018). This method specifically detected *E. faecalis* in the presence of *E. faecium*, and the sensitivity was not affected. This trait is helpful for the clinical identification of pathogenic microorganisms in infected patients.

Evaluation of clinical samples with the RPA-LFS assay demonstrated the same accuracy as qPCR. However, there was no need to extract the DNA separately; only the bacterial liquid after high temperature lysis was needed as a template to complete the reaction. This method could be more useful for the on-site detection of *E. faecium* than existing methods.

## Data availability statement

The original contributions presented in the study are included in the article/**Supplementary Material**. Further inquiries can be directed to the corresponding authors.

## Author contributions

BZ, ZLW, CC and YZL designed the experiments and wrote the manuscript. KW and XJ collected the clinical samples. JH, XLL, SHF, XML, and WJZ performed the experiments. LW and WGZ analyzed the data. All authors reviewed and approved the final version of the manuscript.

## Funding

This study was supported by the Zhenjiang Key R&D Program (Social Development) (SSH20220140238), the Scientific research project of Bengbu Medical College (BYKY2019253ZD), the Jiangsu University 2021 Clinical Medicine Science and Technology Development Fund (Natural Science Category) (JLY2021087), the Lianyungang Second People's Hospital 2020 Hospital Young and Middle-aged Medical Talent Growth Fund (TQ202006), the earmarked fund for CARS-18, Guangxi innovation-driven development project (AA19182012-2), National Key R&D Program of China (2021YFE0111100), Zhenjiang Science and Technology support project (GJ2021015).

## Acknowledgments

We thank International Science Editing (http://www.internationalscienceediting.com) for editing this manuscript.

## Conflict of interest

The authors declare that the research was conducted in the absence of any commercial or financial relationships that could be construed as a potential conflict of interest.

## Publisher’s note

All claims expressed in this article are solely those of the authors and do not necessarily represent those of their affiliated organizations, or those of the publisher, the editors and the reviewers. Any product that may be evaluated in this article, or claim that may be made by its manufacturer, is not guaranteed or endorsed by the publisher.
