# Supplementary material for: Rapid and specific detection of Enterococcus faecalis with a visualized isothermal amplification method
Source: Front Cell Infect Microbiol. 2022 Sep 12;12:991849. doi: 10.3389/fcimb.2022.991849 (PMC9510690; doi:10.3389/fcimb.2022.991849)
Supplement: Supplementary file 1 [file Table_1.docx]

**Supplementary Information**

**Rapid and specific detection of *Enterococcus faecalis* with a visualized isothermal amplification method**

Lei Wang^1,2†^, Xin Jin^2†^, Shihui Fan^2†^, Kun Wang^1^, Wenjun Zhu^1*^, Weiguo Zhao^2*^, Yingzhi Lu^1*^

^1^Department of Oncology & Department of Medicine Laboratory, The Second People's Hospital of Lianyungang (Cancer Hospital of Lianyungang), Lianyungang, China

^2^School of Biotechnology, Jiangsu University of Science and Technology, Zhenjiang, China

^†^These authors contributed equally to this work and share first authorship.

^*^Correspondence:

Wenjun Zhu: [zhuwenjun8812@163.com](mailto:zhuwenjun8812@163.com)

Weiguo Zhao: wgzsri@126.com

Yingzhi Lu : [doctorluyz@126.com](mailto:doctorluyz@126.com)

**Table S1. Detection of clinical samples collected from sputum of patients**

| **Number** | **RPA-LFS** | **qPCR** | **Number** | **RPA-LFS** | **qPCR** | **Number** | **RPA-LFS** | **qPCR** | **Number** | **RPA-LFS** | **qPCR** | **Number** | **RPA-LFS** | **qPCR** | **Number** | **RPA-LFS** | **qPCR** |
| --- | --- | --- | --- | --- | --- | --- | --- | --- | --- | --- | --- | --- | --- | --- | --- | --- | --- |
|  | + | + |  | - | - |  | - | - |  | + | + |  | - | - |  | - | - |
|  | - | - |  | + | + |  | + | + |  |  |  |  | + | + |  | + | + |
|  | - | - |  | - | - |  | - | - |  | + | + |  | - | - |  | - | - |
|  | + | + |  | + | + |  | + | + |  | + | + |  | - | - |  | + | + |
|  | - | - |  | - | - |  | + | + |  | - | - |  | + | + |  | - | - |
|  | - | - |  | + | + |  | - | - |  | - | - |  | + | + |  | + | + |
|  | + | + |  | + | + |  | + | + |  | + | + |  | - | - |  | - | - |
|  | + | + |  | + | + |  | - | - |  | + | + |  | + | + |  | + | + |
|  | + | + |  | - | - |  | + | + |  | - | - |  | - | - |  | - | - |
|  | - | - |  | + | + |  | + | + |  | - | - |  | - | - |  | + | + |
|  | - | - |  | + | + |  | - | - |  | + | + |  | + | + |  | + | + |
|  | - | - |  | - | - |  | - | - |  | - | - |  | - | - |  | - | - |
|  | + | + |  | + | + |  | + | + |  | - | - |  | + | + |  | + | + |
|  | + | + |  | - | - |  | + | + |  | - | - |  | - | - |  | - | - |
|  | - | - |  | + | + |  | + | + |  | + | + |  | - | - |  | - | - |
|  | - | - |  | + | + |  | - | - |  | - | - |  | + | + |  | - | - |
|  | + | + |  | + | + |  | - | - |  | + | + |  | - | - |  | - | - |
|  | - | - |  | - | - |  | - | - |  | + | + |  | - | - |  | - | - |
|  | + | + |  | + | + |  | - | - |  | + | + |  | - | - |  | - | - |
|  | - | - |  | + | + |  | + | + |  | + | + |  | + | + |  | - | - |
|  | + | + |  | - | - |  | - | - |  | - | - |  | - | - |  | + | + |
|  | + | + |  | + | + |  | - | - |  | - | - |  | - | - |  | - | - |
|  | - | - |  | - | - |  | + | + |  | - | - |  | - | - |  | - | - |
|  | + | + |  | + | + |  | - | - |  | - | - |  | - | - |  | - | - |
|  | - | - |  | - | - |  | - | - |  | + | + |  | - | - |  | - | - |
|  | + | + |  | + | + |  | - | - |  | - | - |  | + | + |  | - | - |
|  | - | - |  | - | - |  | - | - |  | + | + |  | - | - |  | - | - |
|  | - | - |  | + | + |  | - | - |  | - | - |  | - | - |  | - | - |
|  | - | - |  | - | - |  | - | - |  | - | - |  | - | - |  | - | - |
|  | - | - |  | - | - |  | - | - |  | + | + |  | - | - |  | + | + |
|  | - | - |  | + | + |  | - | - |  | - | - |  | - | - |  | - | - |
|  | + | + |  | - | - |  | + | + |  | - | - |  | + | + |  | - | - |
|  | - | - |  | + | + |  | - | - |  | + | + |  | - | - |  | - | - |
|  | - | - |  | - | - |  | - | - |  | + | + |  | - | - |  | - | - |
|  | - | - |  | + | + |  | - | - |  | - | - |  | - | - |  | - | - |
|  | - | - |  | - | - |  | - | - |  | - | - |  | - | - |  | - | - |
|  | + | + |  | + | + |  | + | + |  | - | - |  | - | - |  | - | - |
|  | - | - |  | - | - |  | - | - |  | - | - |  | - | - |  | - | - |
|  | - | - |  | + | + |  | - | - |  | - | - |  | - | - |  |  |  |
|  | + | + |  | - | - |  | - | - |  | - | - |  | - | - |  |  |  |
|  | - | - |  | + | + |  | - | - |  | - | - |  | - | - |  |  |  |
|  | - | - |  | - | - |  | + | + |  | - | - |  | - | - |  |  |  |
|  | + | + |  | + | + |  | - | - |  | + | + |  | - | - |  |  |  |
|  | - | - |  | - | - |  | - | - |  | - | - |  | - | - |  |  |  |
|  | - | - |  | + | + |  | - | - |  | - | - |  | - | - |  |  |  |
|  | + | + |  | - | - |  | - | - |  | - | - |  | - | - |  |  |  |
|  | - | - |  | - | - |  | - | - |  | - | - |  | - | - |  |  |  |
|  | - | - |  | - | - |  | - | - |  | - | - |  | - | - |  |  |  |

(+: positive result; -: negative result)
